# Association of *IL-4* and *IL-18* genetic polymorphisms with atopic dermatitis in Chinese children

**DOI:** 10.3389/fped.2023.1202100

**Published:** 2023-05-30

**Authors:** Jianrong Shi, Lin He, Huiwen Zheng, Wei Li, Shuangshuang Huang, Yunling Li, Ran Tao

**Affiliations:** ^1^ Department of Clinical Laboratory, the Children’ s Hospital, Zhejiang University School of Medicine, National Clinical Research Center for Child Health, Hangzhou, China; ^2^ Department of Dermatology, The Children' s Hospital, Zhejiang University School of Medicine, Hangzhou, China

**Keywords:** interleukin-4, interleukin-18, atopic dermatitis, polymorphisms, susceptibility, inflammation

## Abstract

**Background:**

Atopic dermatitis (AD) is a common chronic inflammatory skin disease, adversely affecting nearly 20% of the pediatric population worldwide. Interleukin-4 (IL-4) and interleukin-18 (IL-18) are considered to be involved in the pathogenesis and development of AD. The aim of this study was to investigate the association of *IL-4* and *IL-18* gene polymorphisms with the susceptibility and severity of AD in Chinese children.

**Methods:**

Six candidate single nucleotide polymorphisms (SNPs) in *IL-4* and *IL-18* genes were genotyped through multi-PCR combined with next-generation sequencing in 132 AD children and 100 healthy controls, and all the analyses were performed on blood genome DNA.

**Results:**

The frequencies of G allele, CG genotype and CG + GG genotype of *IL-4* rs2243283, as well as the haplotype *IL-4*/GTT (rs2243283-rs2243250-rs2243248) were all significantly decreased in AD patients compared with the controls [G vs. C: *P* = 0.033, OR = 0.59; CG vs. CC: *P* = 0.024, OR = 0.47; CG + GG vs. CC: *P* = 0.012, OR = 0.49; GTT vs. CCT: *P* = 0.011, OR = 0.65]. Moreover, the frequencies of A allele, AA genotype and AG + AA genotype of *IL-18* rs7106524, along with the haplotype *IL-18*/CAA (rs187238-rs360718-rs7106524) were statistically increased in the severe AD patients (A vs. G: *P* < 0.001, OR = 2.79; AA vs. GG: *P* = 0.003, OR = 5.51; AG + AA vs. GG: *P* = 0.036, OR = 2.93; CAA vs. CAG: *P* = 0.001, OR = 2.86).

**Conclusions:**

Our findings suggested that genetic variation in *IL-4* rs2243283 such as G allele, CG genotype and CG + GG genotype might confer the reduced susceptibility to AD in Chinese children. Furthermore, A allele, AA genotype and AG + AA genotype of *IL-18* rs7106524 explored the strong association with severity in Chinese AD children.

## 1. Introduction

Atopic dermatitis (AD), also known as eczema, is a recurrent inflammatory skin disease that adversely affects a significant proportion of the pediatric population (10%–20%) (1). The quality of life in patients with AD seriously decreased due to the characteristics of intense pruritus, diffuse xerosis and sleep disturbance (2). Although the pathogenesis of AD has not been completely understood, immune imbalance and skin barrier dysfunction led by the interaction of genetic predisposition, immune and microbiome are considered to be the critical causes of AD (1, 3).

It has been confirmed that the immune imbalance between T helper (Th) lymphocytes 1 and 2 can result in abnormal cytokine production, which plays a crucial role in the occurrence and development of immune-mediated and allergic disorders, such as AD, asthma, and allergic rhinitis (4, 5). Interleukin-4 (IL-4), mainly produced by mast cells and activated T cells including Th2 cells, is an important driver of type 2 immunity in these diseases. IL-4 is also considered central to the pathogenesis of AD and key drug target (6). It has been shown that IL-4 acts on both immune cells and histiocytoes, mediating multiple steps of the AD pathogenicity cascade. On the one hand, IL-4 could cause differentiation of naïve CD4^+^ T cells to Th2 cells and induce T cytotoxic 2 (Tc2) cells and group-2 innate lymphoid cells (ILC2) development (6, 7). Through downregulating filaggrin (FLG) expression, IL-4 also amplifies the effects of histamine and stimulates immunoglobulin E (IgE) production by B cells, which could inhibit skin barrier function and induce pruritus or anaphylaxis in skin lesions of acute AD (8, 9). On the other hand, IL-4 regulates the activity of dendritic cells, decreasing their expression of IL-12 and major histocompatibility complex (MHC) class II and increasing IL-10 production, thereby amplifying type 2 inflammation (10). Moreover, mouse model studies have established the centrality of IL-4 in the pathogenesis of AD, highlighting its capability to induce all the histopathological features of AD (6). In summary, the role of IL-4 in the pathogenesis of AD and in mediating a variety of clinical features, including skin inflammation and pruritus, is well established.

Although type 2 inflammation is the dominant immune mechanism, there is growing evidence that AD involves multiple immune pathways. Interleukin-18 (IL-18), identified as an IFN-*γ* inducing factor, is a pro-inflammatory cytokine that plays a critical role in Th1 lymphocytes activation. Moreover, IL-18 synergistically stimulates the production of IgE and Th2 cytokines with IL-12 (11, 12). It has been presented that IL-18 is overexpressed in both AD patients and animal model, suggesting that IL-18 might play a certain role in the pathogenesis and development of dermatitis (13). Recent evidence also demonstrated that IL-18 deficiency alleviated AD-induced lesions by reducing corticotropin-releasing hormone receptor (CRHR) 2 expression in a mouse model (14). Furthermore, Zedan et al. reported that serum IL-18 levels were significantly increased in AD patients and strongly correlated with AD activity, suggesting that IL-18 may help to assess AD activity and therefore would be useful in predicting disease progression (12).

In addition, it has been proposed that the gene polymorphisms of several cytokines are related to immune imbalance in allergic diseases such as bronchial asthma (15). Previous surveys have also indicated that genetic predisposition profoundly affected the onset and severity of AD (16, 17). To our knowledge, several single nucleotide polymorphisms (SNPs) of *IL-4* and *IL-18* genes have been identified their relevance to AD in Macedonian children, Saudi Arabian population, and Egyptians (18–20). Nevertheless, there have been few studies to date on the linkage between these two cytokines polymorphisms and AD in Chinese population. Therefore, in this study, three SNPs of *IL-4* and three SNPs of *IL-18* associated with AD or other allergic diseases in previous researches (19–23) were selected to explore their correlation with the susceptibility and severity of AD in Chinese Han children, so as to assess the population-specific prediction of *IL-4* and *IL-18* genetic variation for AD. This will provide researchers and clinicians with more baseline information including the identifiable risk factors, which may help predict the occurrence and prevent the progression of allergic dermatitis.

## 2. Materials and methods

### 2.1. Subjects

From February 2021 to August 2021, the children with AD attending the dermatology clinic of the Children's Hospital of Zhejiang University School of Medicine were used as the study subjects. Inclusion criteria were determined as follows: newly diagnosed AD patients in accordance with the AD diagnostic criteria of Hanifin and Rajka (1); Age less than 18 years; all subjects and their parents consented to participate in the study. Children with any other systemic inflammatory and autoimmune diseases or a history of medication for skin dysfunctions were excluded. The severity of AD was assessed by the Severity Scoring of Atopic Dermatitis (SCORAD) index (1). In order to observe the association of *IL-4* and *IL-18* SNPs with the severity of AD, AD cases were separated into two subgroups, namely, mild-to-moderate (SCORAD: 0–50) and severe subgroup (SCORAideD: > 50) (1, 24). Meanwhile, one hundred unrelated healthy subjects without a history of atopic or autoimmune disorders were enrolled from the physical examination department in our hospital as controls. All AD cases and controls were of Chinese Han ethnicity. The present study was approved by the Ethics Committee of the Children's Hospital of Zhejiang University School of Medicine in accordance with the principles of the Declaration of Helsinki (2021-IRB-009).

### 2.2. Genotype assessment

One milliliter peripheral blood samples from each subjects were collected in ethylene diamine tetraacetic acid (EDTA) anticoagulant tubes and 200 μl of each specimen was taken for DNA extraction and genotyping. DNA was extracted by Biospin genomic DNA Extraction Kit (BIOER technology, #BSC06S1) and its purity and concentration were detected by Nanodrop1000c spectrophotometer of ThermoScience Company in the United States. The integrity of DNA was observed by 1% agarose gel electrophoresis. The qualified DNA samples were kept at −20 °C for the subsequent genetic analysis.

Three *IL-4* SNPs rs2243283, rs2243250, rs2243248 and three *IL-18* SNPs rs187238, rs360718, rs7106524 were genotyped by multiplex PCR combined with next-generation sequencing technique on X-10 Illumina, a high-throughput genotyping platform of Shanghai BioWing Applied Biotechnology Company (http://www.biowing.com.cn/) (25). The first round PCR was carried out on Gene Amp PCR System 9600 (Norwalk, USA) in the following conditions: 95 °C for 15 min; 4 cycles of 94°C for 30 s, 60°C for 10 min and 72°C for 30 s; 20 cycles of 94°C for 30 s, 60°C for 1 min and 72°C for 30 s. 1 μl of the first round product could be used as a sample for the second round of PCR under these conditions: 95°C for 15 min; 5 cycles of 94°C for 30 s, 60°C for 4 min and 72°C for 30 s; 10 cycles of 94°C for 30 s, 65°C for 1 min and 72°C for 30 s. The detailed primers used for genotyping were shown in Table 1. Then the products of the second round PCR were purified and sequenced on the X-10 platform. All the sequencing data were analyzed by Illumabcl2fastq software.

**Table 1:** Primer sequences for genotyping.

Polymorphic site	Location (GRCh38)	Primer sequence (Forward)	Primer sequence (Reverse)
*IL-4* rs2243283	Chr5:132680901	AGGTGAACAGATTTGGGATATGAC	AATTGAACTCTTGATCTTCTGCTG
*IL-4* rs2243250	Chr5:132673462	TTATGGGTAAGGACCTTATGGAC	TATTTTAACCTGGCTTCTTCCAAG
*IL-4* rs2243248	Chr5:132672952	ACCTTATTGTGTCCACATGAATTC	CTCCCAAAGCTCTGAGATTACAG
*IL-18* rs187238	Chr11:112164265	GAAATAAAGTGGCAGAGGATACG	GGAAGTCTGAAAATGAAGAGAGAC
*IL-18* rs360718	Chr11:112164016	ATGGCTGACTTTCCAAATAAAGAG	GACAGTCAGCAAGGAATTGTCTC
*IL-18* rs7106524	Chr11:112162913	TTGAGAAAGTCTCGCTCTGTTTAG	CTTTCAGGCCAGGTGCACTAG

### 2.3. Statistical analysis

Statistical analysis was performed by the software of Statistical Package for Social Science (SPSS) (version 22.0) (IBM, USA). Continuous variables were shown as mean ± standard deviation (SD), and comparisons between groups were made by *t*-test or Anova variance analysis. Categorical variables were expressed as percentages or ratios. Comparisons between groups were performed using Fisher's exact test or Pearson's chi-square test. Genotypic frequencies of all participants were examined for Hardy-Weinberg equilibrium (HWE) before analysis. HWE, linkage-disequilibrium and haplotypes were achieved via Haploview version 4.2 program (D′ > 0.5 and *r*^2^ > 0.33 indicate strong linkage disequilibrium). All *P* values were bidirectional, and *P* < 0.05 was considered statistically significant. Odds ratio (OR) and 95% confidence intervals (CIs) of different groups or subgroups were calculated using the unconditional logistic regression analysis.

## 3. Results

### 3.1. Demographics

The detailed demographics of 132 AD patients and 100 controls were provided in Table 2. There were 73 males and 59 females with an average age of 2.6 ± 2.1 years in the case group, including 99 (75.00%) mild-to-moderate AD and 33 (25.00%) severe AD. The healthy controls were composed of 66 boys and 34 girls with an average age of 4.9 ± 1.9 years. No significant differences were found in term of gender and age either between the cases and controls, or between the mild-to-moderate subgroup and the severe subgroup.

**Table 2:** Comparison of the demographics between AD patients and the controls.

characteristics	Number	Gender		*P* value	Age	*P* value
		Male [*n* (%)]	Female [*n* (%)]		Years mean ± SD	
Controls	100	66 (66.00)	34 (34.00)	0.131	4.9 ± 1.9	0.232
AD patients	132	73 (55.30)	59 (44.70)		2.6 ± 2.1	
Mild-to-Moderate	99	54 (54.55)	45 (45.45)	0.919	2.6 ± 2.2	0.723
Severe	33	19 (57.58)	14 (42.42)		2.5 ± 1.9	

AD, atopic dermatitis; SD, standard deviation.

### 3.2. Association between genetic polymorphisms and AD susceptibility

The successful genotyping rates of six SNPs were 94.4%–100%. The genotype distributions of all six SNPs in different groups or subgroups were all in agreement with HWE.

As shown in Table 3, the frequency of the mutant G allele of *IL-4* rs2243283 was significantly lower in AD patients comparing to controls (G vs. C: *P* = 0.033, OR = 0.59, 95% CI = 0.38–0.94). Accordingly, we found that carriers with CG genotype of *IL-4* rs2243283 had a statistically decreased risk of AD than carriers with CC genotype (*P* = 0.024; OR = 0.47, 95% CI = 0.27–0.81). Furthermore, the risk of AD susceptibility also reduced when CG and GG genotypes were analyzed together (CG + GG vs. CC: *P* = 0.012, OR = 0.49, 95% CI = 0.28–0.83) (Table 4). No significant differences in allele or genotype frequencies of the other five SNPs were found between AD patients and controls.

**Table 3:** Allele frequencies of six SNPs of *IL-4* and *IL-18* in AD patients and the controls.

Gene	SNPs	Allele	Patients [*n* (%)]	Controls [*n* (%)]	*χ^2^*	*P* value	OR (95% CI)
*IL-4*	rs2243283(C > G)	C	218 (82.58)	148 (74.00)	4.52	0.033*	1.00
		G	46 (17.42)	52 (26.00)			0.59 (0.38–0.94)
	rs2243250 (C > T)	C	46 (17.83)	39 (19.70)	0.15	0.699	1.00
		T	212 (82.17)	159 (80.50)			0.89 (0.55–1.42)
	rs2243248 (T > G)	T	247 (93.56)	187 (93.50)	<0.01	1.000	1.00
		G	17 (6.44)	13 (6.50)			1.01 (0.48–2.13)
*IL-18*	rs187238 (C > G)	C	235 (89.02)	166 (86.46)	0.47	0.495	1.00
		G	29 (10.98)	26 (13.54)			0.79 (0.45–1.39)
	rs360718 (A > C)	A	233 (88.26)	171 (86.36)	0.22	0.641	1.00
		C	31 (11.74)	27 (13.64)			0.84 (0.49–1.47)
	rs7106524 (G > A)	G	133 (50.38)	94 (47.96)	0.18	0.675	1.00
		A	131 (49.62)	102 (52.04)			0.91 (0.63–1.31)

SNP, single nucleotide polymorphism; OR, odds ratio; 95% CI, 95% confidence interval.

* Statistically significant (*P* < 0.05).

**Table 4:** Genotype analysis of *IL-4* rs2243283 in AD patients and the controls.

Genotypes	Patients [*n* (%)]	Controls [*n* (%)]	*χ^2^*	*P* value	OR (95% CI)
CC	90 (68.18)	51 (51.00)	7.42	0.024*	1.00
CG	38 (28.79)	46 (46.00)			0.47 (0.27–0.81)
GG	4 (3.03)	3 (3.00)			0.76 (0.16–3.57)
CG + GG/CC	42 (31.82)	49 (49.00)	6.34	0.012*	0.49 (0.28–0.83)
CC + CG/GG	128 (96.97)	97 (97.00)	<0.01	1.000	1.01 (0.22–4.55)

SNP, single nucleotide polymorphism; OR, odds ratio; 95% CI, 95% confidence interval.

* Statistically significant (*P* < 0.05).

In haplotype analysis, the three SNPs of *IL-4* were located in the same haplotype block with a state of linkage disequilibrium (D′ > 0.5, *r*^2^ > 0.33). Comparing to the haplotype CCT composed of *IL-4* rs2243283, rs2243250 and rs2243248, the frequency of haplotype GTT in AD patients was significantly lower than that in controls (GTT vs. CCT: *P* = 0.011, OR = 0.65, 95% CI = 0.47–0.90) (Table 5). The frequencies of the other two haplotypes CTT and CCG did not differ between AD cases and controls.

**Table 5:** Haplotype frequencies of *IL-4* gene for risk of AD.

Haplotypes (rs2243283, rs2243250, rs2243248)	Patients [*n* (%)]	Controls [*n* (%)]	*χ^2^*	*P* value	OR (95% CI)
CCT	39 (14.77)	26 (13.00)	-	-	1.00
CTT	162 (61.36)	112 (56.00)	0.11	0.744	0.95 (0.72–1.25)
GTT	42 (15.91)	43 (21.50)	6.86	0.011*	0.65 (0.47–0.90)
CCG	9 (3.41)	7 (3.50)	0.02	0.896	0.92 (0.53–1.62)

OR, odds ratio; 95% CI, 95% confidence interval.

* Statistically significant (*P* < 0.05); Haplotypes with the frequencies under 0.05 were not included in haplotype analysis.

### 3.3. Association between genetic polymorphisms and AD severity

As shown in Table 6, the mutant A allele of *IL-18* rs7106524 was associated with a higher risk of severe AD compared with the wild G allele (*P* < 0.001; OR = 2.79, 95% CI = 1.55–5.03). Consistent with the results of allele analysis, the carriers with AA genotype of *IL-18* rs7106524 had an increased risk of severe AD as compared to those with GG genotype (*P* = 0.003; OR = 5.51, 95% CI = 1.77–17.14). Similar risk trends were observed when AA and AG genotypes were analyzed together (AG + AA vs. GG: *P* = 0.036, OR = 2.93, 95% CI = 1.04–8.27) (Table 7). Additionally, the frequency of rs7106524 GG + AG genotype was significantly lower than that in AA genotype (*P* < 0.001; OR = 0.25, 95% CI: 0.11–0.58). No correlation was found between the other five SNPs and the severity of AD.

**Table 6:** Allele frequencies of six SNPs of *IL-4* and *IL-18* in patients with mild-to-moderate and severe AD.

Gene	SNPs	Allele	AD patients		*χ^2^*	*P* value	OR (95% CI)
			Mild-to-Moderate [*n* (%)]	Severe [*n* (%)]			
*IL-4*	rs2243283 (C > G)	C	164 (82.83)	54 (81.82)	0.04	0.851	1.00
		G	34 (17.17)	12 (18.18)			1.07 (0.52–2.22)
	rs2243250 (C > T)	C	39 (19.70)	13 (19.70)	<0.01	1.000	1.00
		T	159 (80.30)	53 (80.30)			1.00 (0.50–2.02)
	rs2243248 (T > G)	T	187 (94.44)	60 (90.91)	0.52	0.469	1.00
		G	11 (5.56)	6 (9.09)			1.70 (0.60–4.79)
*IL-18*	rs187238 (C > G)	C	174 (87.88)	61 (92.42)	1.05	0.306	1.00
		G	24 (12.12)	5 (7.58)			0.59 (0.22–1.63)
	rs360718 (A > C)	A	172 (86.87)	61 (92.42)	1.47	0.225	1.00
		C	26 (13.13)	5 (7.58)			0.54 (0.20–1.48)
	rs7106524 (G > A)	G	112 (56.57)	21 (31.82)	12.13	<0.001*	1.00
		A	86 (43.43)	45 (68.18)			2.79 (1.55–5.03)

SNP, single nucleotide polymorphism; OR, odds ratio; 95% CI, 95% confidence interval.

* Statistically significant (*P* < 0.05).

**Table 7:** Genotype analysis of *IL-18* rs7106524 in patients with mild-to-moderate and severe AD.

Genotype	AD patients		*χ^2^*	*P* value	OR (95%CI)
	Mild-to-Moderate [*n* (%)]	Severe [*n* (%)]			
GG	34 (34.34)	5 (15.15)	11.71	0.003*	1.00
AG	44 (44.44)	11 (33.33)			1.70 (0.54–5.36)
AA	21 (21.21)	17 (51.52)			5.51 (1.77–17.14)
AG + AA/GG	65 (65.66)	28 (84.84)	4.38	0.036*	2.93 (1.04–8.27)
GG + AG/AA	78 (78.79)	16 (48.48)	11.09	<0.001*	0.25 (0.11–0.58)

SNP, single nucleotide polymorphism; OR, odds ratio; 95% CI, 95% confidence interval.

* Statistically significant (*P* < 0.05).

Furthermore, the haplotypes consisting of *IL-18* rs187238, rs360718 and rs7106524 were analyzed for their association with AD severity. The three SNPs of *IL-18* were also located in the same haplotype block with a state of linkage disequilibrium (D′ > 0.5, *r*^2^ > 0.33). As shown in Table 8, the haplotype CAA was related to an increased risk of severe AD contrasted with the wild haplotype CAG (*P* = 0.001; OR = 2.86, 95% CI = 1.49–5.49). Another haplotype GCG did not present a correlation to AD severity.

**Table 8:** Haplotype frequencies of *IL-18* gene in patients with mild-to-moderate and severe AD.

Haplotypes (rs187238, rs360718, rs7106524)	AD patients [*n* (%)]		*χ^2^*	*P* value	OR (95%CI)
	Mild-to-Moderate	Severe			
CAG	83 (41.92)	16 (24.24)	-	-	1.00
CAA	78 (39.39)	43 (65.15)	10.42	0.001*	2.86 (1.49–5.49)
GCG	10 (5.05)	2 (3.03)	<0.01	1.000	1.04 (0.21–5.19)

OR, odds ratio; 95% CI, 95% confidence interval.

* Statistically significant (*P* < 0.05); Haplotypes with the frequencies under 0.05 were not included in haplotype analysis.

## 4. Discussion

AD has always been a hot research topic owing to its high prevalence, recurrence, disability and cost of care (26). Over the past few decades, knowledge of AD has evolved tremendously with insights into pathogenesis, epidemiology, impact of disease, and new therapies (27). Genetic predisposition, epidermal dysfunction, and T-cell driven inflammation are thought to be involved in the complex pathophysiology of AD (1). Among these, genetic predisposition has been reported as the strongest identifiable risk factor and an important contributor influencing the disease incidences and development of AD (1). The current literature has provided evidence for the relationship between genetic polymorphisms of relevant mediators and AD, and thus far, more than 34 AD-related genic causes have been identified, including *FLG*, *IL-13*, *RAD50*, *LRRC32*, *IL2/IL21*, *PRR5l*, *CLEC16A/DEXI*, *ZNF652*, *RUNX3*, *ERBB3*, *IL6R*, *CD207*, *PPP2R3C*, *IL-7R*, *STAT3*, *ZBTB10* and *TLR4*, etc (1, 16, 17).

In this case-control study, the results demonstrated the association of *IL-4* gene polymorphisms with the susceptibility to AD in Chinese Han children. The data showed that the mutant G allele, CG genotype and CG + GG genotype of *IL-4* rs2243283 were associated with a significantly reduced risk of AD. We also found a correlation between *IL-4*/GTT haplotype composed of rs2243283, rs2243250, rs2243248 and lower AD risk. As reported, rs2243283 gene variants have been confirmed to correlate to some chronic inflammatory and immune diseases, such as asthma, chronic periodontitis, and autoimmune hepatitis (22). However, as we know, rare studies focused on the linkage of this SNP with AD. Therefore, our data might provide a new risk predictor for estimating AD predisposition. Except for rs2243283, the other two SNPs of *IL-4* had no relationship with AD in the present study. *IL-4* rs2243250 (−590C/T) is a common locus in researches on the association of *IL-4* gene polymorphisms with AD. But the findings regarding the correlation between *IL-4* rs2243250 and AD susceptibility are controversial. Consistent with our results were the investigations conducted in the Saudi and Macedonian pediatric population (18, 19). Inconsistent with our data were the surveys carried out among Czech children and another subset of Chinese pediatric participants (28, 29). Chinese academic Shang H reported that the 590 T and 589 T alleles may be related to the increased risk of AD, based on a case-control study that enrolled 82 AD patients and 100 healthy controls (29). Whether the discrepancy between our data and the above result is due to different sample sizes remains to be clarified by further investigations with larger numbers of participants. The contradictions in these findings also might be attributed to different genetic backgrounds and environments (30). The larger studies in populations with the same ethnic background are needed to confirm or refute these results. Additionally, no link of *IL-4* genetic polymorphisms to AD severity was found in this study. With the exception of one report published in 2002 that indicated the *IL-4* 590 T allele was associated with the severity of AD (31), several researches in recent years showed no relevance between *IL-4* rs2243250 and AD severity (29, 30).

Besides the etiology diagnosis of AD, studies on prediction of the AD severity remains challenging. Several inflammation-related gene polymorphisms are attached with the severity of AD and could be used to predict potential severity (16, 32, 33). IL-18 is a glycoprotein encoded by the *IL-18* gene, which has been mapped to chromosome 11q22.2-q22.3 (34). And its length is approximately 21.61 kb (34). Although serum level of IL-18 was regarded as a possible biomarker for the severity of AD (35), there was few publications so far on the association between *IL-18* genetic variations and AD severity. The present study was conducted for the first time in Chinese Han children and revealed the relation of *IL-18* polymorphisms to AD severity. Our data explored that A allele, AA genotype and AG + AA genotype of *IL-18* rs7106524, as well as *IL-18*/CAA haplotype consisting of rs187238, rs360718 and rs7106524 were associated with a high risk of severe AD, suggesting the genetic polymorphisms of *IL-18* rs7106524 might be used as potential predictors of AD severity. Consequently, the results may be useful in making appropriate therapy recommendations and preventing dermatitis development. Nevertheless, the functions of the investigated polymorphisms are still not well elucidated, so further studies are still needed to explore the potential molecular mechanisms underlying the association between *IL-18* rs7106524 polymorphism and AD severity.

In addition, no linkage between *IL-18* genetic polymorphisms and susceptibility to AD was found, inconsistent with several previous investigations. For example, the merged quantitative analysis in a recent meta-analysis study displayed that *IL-18* rs187238 polymorphisms might affect the risk of AD in overall population (36). This discrepancy between studies might still be due to the sample size and ethnicity. Future multicenter studies with a large sample size will be carried out to further explore the relationship between genetic variation in cytokines and AD. Besides, whether nucleotide polymorphisms in *IL-4* and *IL-18* genes affect alterations in amino acid sequences and protein secondary structure, and whether they subsequently influence expression levels and protein functions, deserve deeper consideration and require prospective work to elucidate. Perhaps, in-depth cell-specific transcriptomic investigation will guide better understanding of cellular impact and underlying mechanisms of the IL-4 and IL-18 signaling pathways, and then lead to better drug targets for AD treatment.

## 5. Conclusion

In summary, the results of the current study revealed that the CG genotype of *IL-4* rs2243283 and the *IL-4*/GTT haplotype confer the decreased susceptibility to AD in Chinese pediatric population. And *IL-18* rs7106524 AA genotype and the *IL-18*/CAA haplotype might serve as predictors of high risk for severe AD in Chinese children.

## Data Availability

The data presented in the study are deposited in the figshare repository, accession number https://doi.org/10.6084/m9.figshare.22776002.v1.
